# Extracellular Vesicles from Thapsigargin-Treated Mesenchymal Stem Cells Ameliorated Experimental Colitis via Enhanced Immunomodulatory Properties

**DOI:** 10.3390/biomedicines9020209

**Published:** 2021-02-18

**Authors:** Hansol Joo, Mi-Kyung Oh, Ji Yeon Kang, Hyun Sung Park, Dong-Hoon Chae, Jieun Kim, Jong-Hee Lee, Hee Min Yoo, Uimook Choi, Do-Kyun Kim, Hakmo Lee, Sungjoo Kim, Kyung-Rok Yu

**Affiliations:** 1 Department of Biomedicine and Health Sciences, College of Medicine, The Catholic University of Korea, Seoul 06591, Korea; so1hanssol@gmail.com (H.J.); ipomk@catholic.ac.kr (M.-K.O.); acme807@gmail.com (J.Y.K.); hyunsungp@gmail.com (H.S.P.); jieun_lyn@naver.com (J.K.); 2 Department of Medical Life Sciences, College of Medicine, The Catholic University of Korea, Seoul 06591, Korea; 3 Department of Agricultural Biotechnology, Research Institute of Agriculture and Life Sciences, Seoul National University, Seoul 08826, Korea; chaedh96@naver.com; 4 National Primate Research Center (NPRC), Korea Research Institute of Bioscience and Biotechnology (KRIBB), Cheongju 28116, Korea; jonglee@kribb.re.kr; 5 Biometrology Group, Korea Research Institute of Standards and Science (KRISS), Daejeon 34113, Korea; hmy@kriss.re.kr; 6 Laboratory of Clinical Immunology and Microbiology, NIAID, NIH, Bethesda, MD 20892, USA; uchoi@niaid.nih.gov; 7 Center for Biomolecular and Cellular Structure, Institute for Basic Science (IBS), Daejeon 34126, Korea; kdkwhite@hanmail.net; 8 Design One Health Inc., Guri 11906, Korea; ehakmo@gmail.com

**Keywords:** EVs, mesenchymal stem cells, thapsigargin, immunomodulatory property, indoleamine 2,3-dioxygenase (IDO), colitis model

## Abstract

Therapeutic applications of extracellular vesicles (EVs) derived from mesenchymal stem cells (MSCs) have attracted considerable attention because of their immunomodulatory properties against immune-mediated, inflammatory diseases. Here, we demonstrated enhanced immunomodulatory properties of EVs secreted from endoplasmic reticulum (ER) stress inducer thapsigargin (TSG)-primed human Wharton’s jelly-derived MSCs (WJ-MSCs). EVs from TSG-primed WJ-MSCs (TSG-EV) showed increased yield and expression of immunomodulatory factors, such as transforming growth factor-β1 (TGFβ), cyclooxygenase-2 (COX2), and especially indoleamine 2,3-dioxygenase (IDO), compared to control EVs. TSG-EV showed a significantly enhanced immunosuppressive effect on human peripheral blood-derived T cell proliferation and Th1 and Th17 differentiation, whereas Treg and M2-type macrophage were enriched compared to a control EV-treated group. Furthermore, TSG-EV substantially mitigated mouse experimental colitis by reducing the inflammatory response and maintaining intestinal barrier integrity. A significant increase of Tregs and M2-type macrophages in colitic colons of a TSG-EV-treated mouse suggests an anti-inflammatory effect of TSG-EV in colitis model, possibly mediated by Treg and macrophage polarization. These data indicate that TSG treatment promoted immunomodulatory properties of EVs from WJ-MSCs, and TSG-EV may provide a new therapeutic approach for treatment of colitis.

## 1. Introduction

Previous reports demonstrated promising therapeutic effects of mesenchymal stem cells (play an important role in immune regulation by mediating the delivery of secreted MSCs) through immunosuppressive, angiomodulatory, and paracrine factor activities [1,2,3]. MSC paracrine secretome plays important immunomodulation in both the innate and the adaptive immune system by suppression of CD4 and CD8 T cells activation/proliferation and pro-inflammatory type 1 helper T cells (Th1) [4]. MSCs are also known to switch activated inflammatory M1-type macrophage to an M2-type macrophage [5]. We and others have shown that a diverse repertoire of cytokines and chemokines modulate the function of immune cells including prostaglandin E2 (PGE2), indoleamine 2,3-dioxygenase (IDO), nitric oxide, transforming growth factor-beta (TGF-β), IL-6, and IL-10 [6]. Among the MSC secretome, MSC-derived extracellular vesicles (EVs) have recently attracted attention. EVs are lipid bilayer vesicles secreted by cells into the extracellular matrix and play an important role in cell-to-cell communication. The three main classes of EVs are exosomes, microvesicles, and apoptotic bodies [7,8,9,10]. MSC EVs promote activation of immune regulatory pathways by transferring proteins, mRNAs, microRNAs (miRNAs), and other components to target cells [11]. The function and cargo of EVs differ according to cell type, and cell stress can affect the cargo of EVs [12].

Therapeutic effects of MSC EVs have been studied to target autoimmune uveitis, inflammatory lung diseases, graft-versus-host disease, and liver fibrosis [13,14,15,16]. Recently, studies have reported that injection of EVs into a DSS-induced colitis mouse model ameliorated colon inflammation [17,18]. In addition, UC-MSC-derived EVs have been shown to reduce colitis in mice by repolarizing macrophages to an anti-inflammatory M2 phenotype [19]. An et al. reported that TNFα- and IFNγ-primed MSC-derived EVs effectively induce macrophage polarization with an anti-inflammatory M2 phenotype and suppress activated immunity by enhancing regulatory T cells in the inflamed mouse colon [20]. Taken together, these findings are evidence that EVs secreted from MSCs elicit therapeutic effects of MSCs and may be further enhanced by pretreatment and conditioning.

The newly synthesized proteins undergo folding and translocation through the endoplasmic reticulum (ER) membrane and ER plays an important role in coordinating signaling pathways to ensure cellular homeostasis. However, under stressful pathological and physiological conditions, when ER is unable to maintain homeostasis, the unfolded protein response (UPR) is activated and may have an impact on cellular function [21,22,23]. Prolonged dysfunction and stress in the ER is involved in the pathogenesis of many diseases, including metabolic diseases and inflammation-related diseases [24]. Thapsigargin (TSG) increases cytosolic calcium concentration and depletes ER calcium stores, leading to ER stress. Interestingly, induction of calcium homeostasis/ER stress or treatment with TSG is closely related to the yield and function of EVs. Savina et al. reported that transferrin stimulated EV release in a calcium-dependent manner in K562 cells [25]. Severe ER stress induced by tunicamycin or TSG in BeWo choriocarcinoma cells lead to release of extracellular vesicles carrying pro-inflammatory damage-associated molecular pattern (DAMP) molecules [26]. Xia et al. reported that TSG-stimulated MSCs showed enhanced inhibition of rheumatoid arthritis follicular helper-like T cells through prostaglandin E2 (PGE2) [27]. However, therapeutic effects of EVs derived from TSG-treated MSCs have not been reported. Although the stimulation of EV release under ER stress condition have been consistently reported, immunomodulatory properties of stimulated EVs are in conflict with each other according to cell type and the concentration of ER stress inducer.

Here, we investigated the immunomodulatory properties of EVs released from TSG-treated WJ-MSCs on activation and polarization of human T cells and macrophages. In addition, we examined the therapeutic efficacy of TSG-primed EVs in murine colitis and examined the possible biological mechanism underlying the therapeutic value of EVs.

## 2. Materials and Methods

### 2.1. Preparation and Culture of WJ-MSCs

For the isolation of Wharton’s jelly-derived mesenchymal stem cells (WJ-MSCs), Wharton’s jelly tissue was obtained from the umbilical cords after normal full-term delivery with the written informed consent of the mothers and the approved by the Asan Medical Center Institutional Review Board (protocol no. 2015–3030). The WJ-MSCs were isolated as previously described [28]. In this study, MSCs in 15–22 passages were used. MSCs were cultured in DMEM (Gibco BRL, Grand Island, NY, USA) with 10% FBS (Tissue Culture Biologicals, Tulare, CA, USA), 1% Glutamax (Gibco), 25 ng/mL EGF (Biolegend NS, Inc., San Diego, CA, USA), 50 ng/mL bFGF (Peprotech, Rocky Hill, NJ, USA), and 1% antibiotic/anticotic (Gibco) at 37 °C in 5% CO_2_. Upon reaching 80~90% confluence, cells were detached with 0.05% Trypsin-EDTA (Gibco) for further subcultures.

### 2.2. Cell Viability Assay

WJ-MSCs were cultured in 24-well cell plate at a density of 2 × 10^5^ for overnight and treated with Thapsigargin (TSG, 0, 0.1, 0.5, 1, 5, 10 μM) (Sigma-Aldrich, St. Louis, MO, USA). After 24 h incubation, 0.5 mg/mL MTT (3-(4,5-dimethylthiazol-2-yl)-2,5-diphenyltetrazolium bromide) solution (Sigma-Aldrich) were added in the medium and incubated for an additional 4 h. The crystals were dissolved with lysis buffer (0.04 M HCl in isopropanol) and absorbance was measured at 570 nm using a spectrophotometer (Biotek, Winooski, WT, USA).

### 2.3. Isolation and Characterization of EVs

WJ-MSC EVs were prepared by ultracentrifugation as previously described [29]. Briefly, the cells (3 × 10^6^) were cultured overnight in 100 mm culture dish, and then stimulated with 1 μM TSG (Sigma-Aldrich) for 24 h. The medium was then replaced with medium supplemented with 10% EV-depleted FBS. EV-depleted FBS was obtained by ultracentrifugation at 100,000× *g* for 18 h. After 72 h incubation, the culture medium was collected and centrifuged at 300× *g* for 10 min, 2500× *g* for 25 min, 10,000× *g* for 30 min at 4 °C. The supernatant was filtered through 0.22 µm filters and ultracentrifuged at 100,000× *g* for 2 h. Then, the EV pellet was washed by centrifugation in sterile PBS at 100,000× *g* for 2 h, suspended in 200 μL of PBS and stored at −80 °C until use. Although this protocol aims to isolate exosomes with microvesicle elimination step, we used the term “EV”, rather than “exosomes” to follow the nomenclature recommendation by the International Society for Extracellular Vesicles (ISEV) [30]. For inhibition of EVs generation, cells were treated with culture medium containing 10 μM GW4869 (Santa Cruz Biotechnology, Dallas, TX, USA) for 12 h. The medium was then replaced with medium supplemented with 10% EV-depleted FBS. After 72 h, EVs from the culture supernatants were isolated using ultracentrifugation, and stored at −80 °C until use (concentrated-CM). For all mouse experiments, concentrated-CM derived from GW4869 treated MSCs (inhibition of EV secretion) were used as a vehicle control. The total protein concentration of EVs was determined by the BCA assay kit (Thermo Fisher Scientific, Waltham, MA, USA). EV size and number was determined by qNano (Izon Science, Christchurch, New Zealand) and transmission electron microscopy (TEM; Jeol, Tokyo, Japan) according to the manufacturer’s instructions. EV-specific marker (CD63, System Bioscience, Palo Alto, CA, USA) was measured by Western blot analysis.

### 2.4. Western Blot Analysis

WJ-MSC lysates and EVs were lysed with RIPA buffer (Thermo Fisher) with protease inhibitor (Thermo Fisher). Equal amounts of proteins or EV were subjected to SDS-PAGE and analyzed with primary antibodies: rabbit polyclonal CD63 (EXOAB-CD63A-1, System Bioscience), rabbit polyclonal COX2 (ab15191, Abcam, Cambridge, UK), mouse monoclonal IDO (clone 1F8.2, MAB10009, Merk Millipore, Darmstadt, Germany), rabbit polyclonal TGFβ1(ab92486, Abcam), rabbit monoclonal GRP78 (clone C50B12, #3177, Cell signaling technology, Danvers, MA, USA), mouse monoclonal CHOP (clone L63F7, #2895, Cell signaling technology), mouse monoclonal GAPDH (clone 0411, sc-47724, Santa Cruz). The bands were detected using enhanced chemiluminescence (Thermo Fisher) and images were captured using LAS-3000 system (Fujifilm, Tokyo, Japan).

### 2.5. Quantitative RT-PCR

Total RNA was extracted from cells, EVs, or tissue samples using TRizol reagent (Invitrogen, Waltham, MA, USA), and cDNA was synthesized from total RNA using Superscript™ III reverse transcriptase (Invitrogen). Quantitative PCR was measured using the SYBR Green Master Mix (Applied Biosystems, Foster City, CA, USA) in MX300P thermal cycler (Stratagene, San Diego, CA, USA). The expression levels of mRNA were normalized to that of GAPDH. The primer sequences for qRT-PCR are listed in Supplementary Table S1.

### 2.6. Apoptosis Assay

Mononuclear cells (MNCs) were isolated from hUCBs using Ficoll-Hypaque (GE Healthcare, Chicago, IL, USA) gradient centrifugation as described previously [31], and resuspended in RPMI 1640 medium supplemented with 10% Exo-depleted FBS. Umbilical cord blood (UCB) units were obtained from the Catholic Hematopoietic Stem Cell Bank (CHSCB) in Korea from April 2019 to June 2020 under the institutional review board approval (IRB No.2019-0467-0003). MNCs were stimulated with 5 μg/mL Con A (Concanavalin A) and seeded in 96 well plate (1 × 10^5^/well) in growth medium with and without EVs (4 μg) derived from naive or TSG primed WJ-MSCs for 72 h. Annexin V/7AAD (BD Biosciences, Franklin Lakes, NJ, USA) staining was performed to determine cell apoptosis according to the manufacturer’s instructions and analyzed by flow cytometry.

### 2.7. PBMC Isolation

Human peripheral bloods from healthy donors were provided from the Korean Red Cross Blood Services (Seoul, Korea) under approval of the Institutional Review Board (IRB) of the Catholic University (IRB No.2019-2891-0003). Peripheral blood mononuclear cells (PBMCs) were isolated by centrifugation over a Ficoll-Hypaque density gradient as described previously [32], and resuspended in RPMI 1640 medium supplemented with 10% Exo-depleted FBS.

### 2.8. Cell Proliferation Assay

The proliferation of T cells was quantified by CFSE dilution. Isolated PBMCs were labeled with 2 μM carboxyfluorescein diacetate succinimidyl ester (CFSE; CellTrace CFSE Cell Proliferation Kit, Thermo Fisher Scientific). CFSE-labeled PBMCs (1 × 10^5^/well) were cultured in 96 well plate with or without EVs (4 μg) derived from naive or TSG primed WJ-MSCs, in the presence of anti-CD3/CD28 microbeads (Gibco) and recombinant human IL-2 (30 U/mL, Peprotech). After 6 days of incubation, the cells were stained with human monoclonal antibodies against CD45-APC-H7 (clone 2D1), CD3-PE-Cy5 (clone HIT3α), CD4-FITC (clone RPA-T4), and CD8-BV421 (clone RPA-T8, BD Biosciences) and measured by flow cytometry. All cells were gated on 7AAD negative cells.

### 2.9. The Cell Differentiation

CD4^+^ T cells isolation from PBMCs lymphocytes were performed using human CD4 T Cell isolation kit (Miltenyi Biotec, Marburg, Germany) according to the manufacturer’s protocol. CD4^+^ T cells were stimulated with of anti-CD3/CD28 microbeads and IL-2 (20 ng/mL) and added with specific cytokine for the differentiation of specific Th cells; IFNγ (25 ng/mL) and IL-12 (25 ng/mL) for Th1; IL-6 (50 ng/mL) and TGFβ (25 ng/mL) for Th17; TGFβ (25 ng/mL) and Retinoic acid (10 nM) for Treg. Cytokine-treated CD4^+^ T cells (5 × 10^5^/well) were cultured for 5 days by adding EVs derived from naive or TSG primed WJ-MSCs in a 24 well plate. For intracellular staining, cells were stimulated with a cell stimulation cocktail (Invitrogen) for 5 h before staining. The cells were stained with cell surface marker anti-CD3-PE-Cy5, anti-CD4-FITC and anti-CD25-APC (clone M-A251, BD Biosciences) antibodies and then fixed/permeabilized and stained with fluorescence-labeled anti-IFNγ-APC (clone 4S.B3), anti-IL-17A-PE (clone SCPL1362), and anti-Foxp3-PE (clone 259D/C7, BD Biosciences) antibodies. Staining cells were analyzed by flow cytometry.

### 2.10. Differentiation and Polarization of Human Macrophage

CD14^+^ monocytes were isolated from PBMCs lymphocytes using CD14 conjugated microbeads (Miltenyi Biotec). To generate macrophages, monocytes (5 × 10^5^/well) were cultured for 6 days in RPMI 1640 medium (Gibco) containing 10% FBS with GM-CSF (50 ng/mL; Peprotech) or M-CSF (100 ng/mL) in a 24 well plate. For M1 polarization, GM-CSF derived macrophages were stimulated with IFNγ (20 ng/mL) plus LPS (1 μg/mL) in presence of EVs derived from naive or TSG primed WJ-MSC for 48 h. For M2 polarization, M-CSF derived macrophages were stimulated with IL-4 (20 ng/mL) plus IL-13 (20 ng/mL) in presence of EVs derived from naive or TSG primed WJ-MSCs for 48 h. After co-culture, the cells were stained with human monoclonal antibodies against CD14-APC-Cy7 (clone MφP9), CD80-APC-R700 (clone L3307.4), CD86-BV650 (clone 2331), CD206 (clone 19.2), and CD163- BV421 (clone GHI/61. BD Biosciences) and analyzed by flow cytometry.

### 2.11. DSS-Induced Colitis Mice

Acute colitis in 7-weeks-old C57BL/6 mice was induced by the administration of 3% (*w*/*v*) dextran sulfate sodium (DSS; MP Biomedicals, Santa Ana, CA, USA) in the drinking water for day 7. Mice were randomly divided into the four groups of 10 mice per group: (1) Control (negative control), (2) DSS with concentrated-CM derived from GW4869 treated MSCs (positive control), (3) DSS with EVs derived from naive MSC, (4) DSS with EVs derived from TSG primed MSC. At days 1, 3, and 5, mice were intraperitoneally injected with 200 μg EV diluted in 200 μL PBS. Mice were euthanized at day 10. The severity of colitis was assessed daily by utilizing the disease activity index (DAI), including body weight loss (0–4), stool consistency (0–4), stool blood (0–4), coat roughness (0–4), rectal prolapse (0–3), hunched posture (0–3), bedding contamination (0–2), and not inquisitive/alert (0–2). All animal experimental procedures were approved by the Animal Research Ethics Committee of the Catholic University of Korea (IACUC no. 2019-0301-03).

### 2.12. Histopathologic Evaluation

On day 10, mice were sacrificed and colon length was measured. The colon tissues were fixed in 4% formaldehyde (Wako, Osaka, Japan), embedded in paraffin, and sectioned at 5 µm. For histological analysis, colon sections were stained with hematoxylin and eosin (H&E). Histopathology scores were determined in a blinded manner by the degree of inflammatory cell infiltration (0–4) and the degree of tissue injury (0–4).

### 2.13. Isolation of Cells from the Lamina Propria of Colons in Mice

Lamina propria cells were isolated from the intestine as previously described [18]. The colon tissue was cut into small pieces (3 mm × 3 mm) and incubated with HBSS buffer containing 2 mM EDTA (Sigma) in a shaking incubator at 220 rpm for 30 min at 37 °C to remove the epithelium. Then tissue was digested in RPMI containing 5% FBS, 1 mg/mL Collagenase D (Sigma) and 0.1 mg/mL DNase I (Sigma) in a shaking incubator at 220 rpm for 60 min at 37 °C. The digestion samples were filtered through a 40 μm cell strainer and then were centrifuged for 5 min at 300× *g*. The isolated cells were stained with anti-mouse CD11b-BV480 (clone M1/70), anti-mouse F4/80-BV421 (clone T45-2342), anti-mouse CD206-Alexa Flour 647 (clone MR5D3) and anti-mouse arginase1-PE-Cy7 (clone A1exF5, BD Biosciences) and analyzed by flow cytometry.

### 2.14. Myeloperoxidase (MPO) Activity Assay

Infiltration of neutrophils into the colon tissue was measured with the MPO Colorimetric Activity Assay Kit (Sigma). Briefly, the colon was homogenized in MPO assay buffer and supernatants were collected by centrifugation at 13,000× *g* for 10 min. The supernatant was mixed with MPO assay buffer and MPO substrate, incubated at 25 °C for 120 min, then tetramethylbenzidine (TNB) probe was added. Absorbance was measured at 412 nm using a spectrophotometer (Biotek).

### 2.15. Statistical Analysis

Statistical analysis was performed using GraphPad Prism v8.0.1 (GraphPad Software, San Diego, CA, USA) with one-way analysis of variance (ANOVA) followed by Tukey’s multiple comparisons test. All data were presented as the mean ± S.D. *p*-value < 0.05 were considered to indicate statistically significant.

## 3. Results

### 3.1. Induction of ER Stress by TSG Stimulates the Release of EV on WJ-MSCs

**Figure 1:**
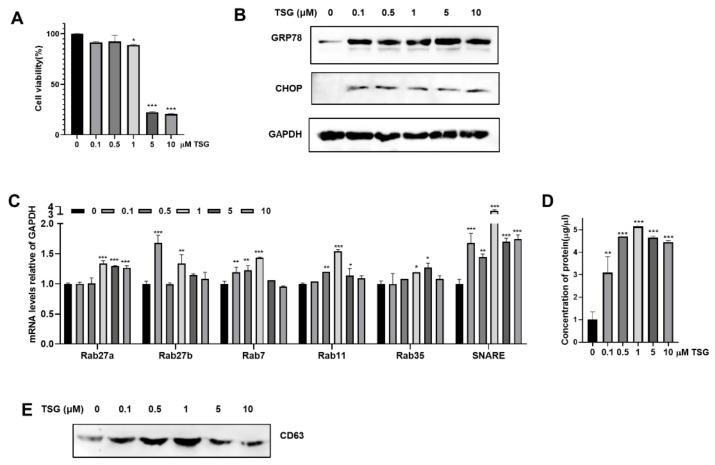
Induction of ER stress by TSG stimulates EV release in WJ-MSCs. (**A**–**C**) WJ-MSCs were treated with increasing doses of TSG (0, 0.1, 0.5, 1, 5, 10 μM) for 24 h. (**A**) Cell viability was assessed by MTT assay. (**B**) The expression of GRP78 and CHOP in cell lysates (40 μg) was measured by Western blot analysis. (**C**) Gene expressions of Rab27a, Rab27b, Rab7, Rab11, Rab35, and SNARE in cell lysates was detected by qRT-PCR. (**D**,**E**) EVs derived from TSG treated WJ-MSCs were isolated by standard ultracentrifugation. (**D**) Protein concentration of EVs was measured by BCA assay. (**E**) Expression of CD63 EV marker in same volume (15 μL) of EV fraction was assessed by Western blot. Data are represented as the means ± S.D. of three independent experiments (* *p* < 0.05, ** *p* < 0.01, *** *p* < 0.001).

WJ-MSCs were isolated and cultured as previously described [6] and further characterized by surface marker expression profile. Flow cytometric analysis demonstrated that WJ-MSCs positively expressed MSC-specific cell surface markers CD29, CD44, CD73, CD105, and CD146 and negatively expressed hematopoietic stem cell specific markers CD34 and CD45 (Supplementary Figure S1A). To investigate the effects of ER stress on EV release of WJ-MSCs, we induced ER stress with TSG in WJ-MSCs. WJ-MSCs were treated with increasing doses of TSG, and cell viability was determined after 24 h. WJ-MSCs treated with TSG up to 1 μM showed no or minimal changes on cell viability, but higher concentrations of TSG (5 μM or 10 μM) significantly reduced cell viability (Figure 1A). The ER stress markers, GRP78 and CHOP expressions were significantly increased after TSG treatment in WJ-MSCs (Figure 1B). Expressions of Rab27a, Rab27b, Rab7, Rab11, Rab35, and SNARE, known regulators of EV release, were significantly increased with TSG treatment (Figure 1C).

Next, we investigated the effect of TSG on EV release. EVs were isolated from the culture supernatant by standard ultracentrifugation methods. (Supplementary Figure S1B). We found that TSG treatment significantly increased total protein concentration of EVs secreted by WJ-MSCs, with 1 μM TSG showing the greatest effect on EV production (Figure 1D). Consistent with these findings, the expression of the EV marker CD63 in same volume of EV fraction was significantly increased in 1 μM TSG treatment (Figure 1E).

### 3.2. Characterization of EVs Derived from TSG Primed WJ-MSCs

**Figure 2:**
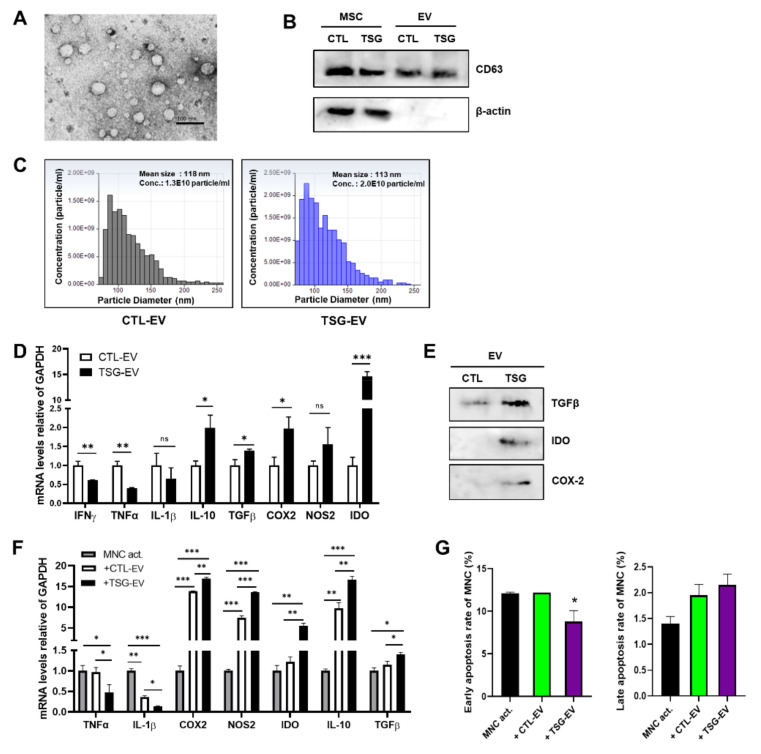
Characterization and immunomodulatory factors of EVs derived from naive or TSG primed WJ-MSC. EVs derived from naive or TSG primed WJ-MSCs were isolated by ultracentrifugation. (**A**) The transmission electron microscopy (TEM) image of EVs. Scale bar, 100 nm. (**B**) Western blot analysis of CD63 and β-actin (10 μg) in WJ-MSCs and EVs. (**C**) Size distribution and concentration of EVs by qNano. Data shown in (**A**–**C**) are representative of independent experiments repeated three times. (**D**) Relative mRNA expressions of the immunomodulatory factors in CTL-EV and TSG-EV were determined by qRT-PCR. (**E**) The expression of TGFβ, IDO and COX2 in CTL-EV and TSG-EV lysates (9 μg) were measured by Western blot analysis. (**F**) Con A-stimulated MNCs were co-cultured with CTL-EV and TSG-EV for 48 h. Gene expression of immunomodulatory factor were determined by qRT-PCR. (**G**) After 72 h treatment with CTL-EV and TSG-EV, Con A-stimulated MNCs were stained with Annexin V and 7AAD and analyzed by flow cytometry. Data are represented as the means ± S.D. of three independent experiments (* *p* < 0.05, ** *p* < 0.01, *** *p* < 0.001).

EVs were isolated from the culture supernatant of naive (CTL-EV) or TSG-primed WJ-MSCs (TSG-EV) by standard ultracentrifugation methods. Transmission electron microscopic analysis showed that isolated EVs include round vesicles of 50–120 nm in diameter (Figure 2A). The EV-specific marker CD63 was expressed in CTL-EV and TSG-EV, but cytoplasmic marker β-actin was not expressed in either EV (Figure 2B). qNano analysis was performed to evaluate the size distribution of EVs, and the mean particle diameter was 110–120 nm in both CTL-EV and TSG-EV (Figure 2C). Interestingly, production of EVs derived from TSG-primed WJ-MSCs was higher than that of EVs derived from naive WJ-MSCs (Figure 2C).

As pro- and anti-inflammatory cytokine secretion pattern determines the immunomodulatory properties of MSCs or MSC-derived EVs, we examined the expression of inflammatory genes. TSG-EV had significantly higher expression levels of IL-10, TGFβ, COX2, and IDO compared to CTL-EV but lower expression of pro-inflammatory cytokines including IFNγ, TNFα, and IL-1β (Figure 2D). Consistent with the mRNA expression results, TGFβ, COX2, and IDO protein expression levels were significantly increased in TSG-primed EVs (Figure 2E).

### 3.3. TSG-Primed EVs Inhibit T Cell Proliferation by Increasing Anti-Inflammatory Cytokine Production

**Figure 3:**
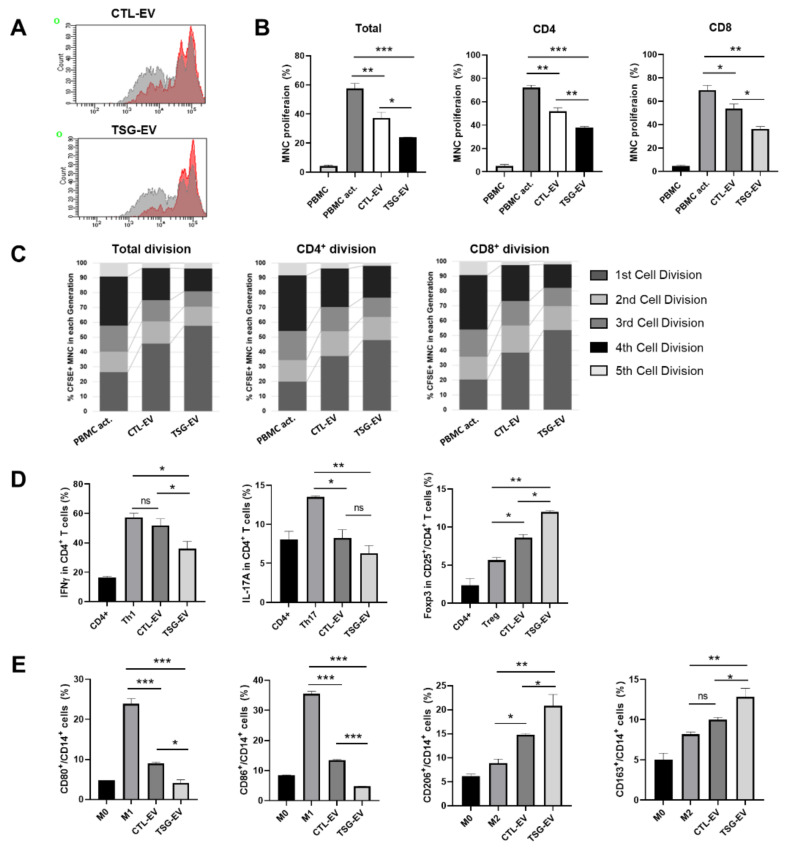
EVs from TSG-primed WJ-MSCs regulate T cell proliferation and T helper (Th) cell differentiation and macrophage polarization. (**A**–**C**) CTL-EV and TSG-EV were co-cultured with CFSE-labeled PBMCs stimulated by anti-CD3/28 beads and IL-2. After 6 days, proliferation of T cell was measured by flow cytometry analysis. (**A**) Representative histogram of total T cells proliferations. The flow cytometry histograms show representative analyzes from three independent experiments. (**B**) Quantitative analysis of proliferation rates of the total T, CD4^+^ T, and CD8^+^ T cells. (**C**) Cell division in the five individual parts was tracked by the fluorescence profile of CFSE-labeled cells. (**D**) CD4^+^ T cells were incubated with specific lineage-driving cytokines with or without CTL-EV and TSG-EV in the presence of anti-CD3/CD28 beads and IL-2 for 5 days. The percentage of CD4^+^IFNγ^+^Th1, CD4^+^IL-17A^+^Th17, CD4^+^CD25^+^Foxp3^+^Treg cell was analyzed by flow cytometry and quantified. (**E**) CD14^+^ monocytes were differentiated into macrophage by GM-CSF or M-CSF for 6 days. Macrophages were activated with either the M1 cytokines (M0-GM; IFNγ + LPS) or M2 cytokines (M0-M; IL-4 + IL-13) for 48 h, respectively. Expression of M1 (CD80, CD86) and M2 (CD206, CD163) macrophage surface markers was analyzed by flow cytometry and quantified. The data are shown as the mean ± S.D. of three independent experiments (* *p* < 0.05, ** *p* < 0.01, *** *p* < 0.001).

To confirm the immunomodulatory properties of TSG-treated WJ-MSCs, we co-cultured CFSE-labeled PBMCs with WJ-MSCs in the presence of anti-CD3/CD28 beads and IL-2. TSG-treated WJ-MSCs showed enhanced immunosuppressive effect confirmed by total T, CD4^+^ T, and CD8^+^ T cell proliferation compared to naive WJ-MSCs (Supplementary Figure S1C). To evaluate the immunomodulatory properties of TSG-primed EVs, the expression levels of anti-inflammatory cytokines and pro-inflammatory cytokines were measured in activated mononuclear cells (MNCs). When concanavalin A-stimulated MNCs were treated with EVs, the expression levels of COX2, NOS2, IDO, IL-10, and TGFβ increased, while the expression levels of TNFα and IL-1β decreased. This effect was more prominent in activated MNCs treated with TSG-EV than in those treated with CTL-EV (Figure 2F). TSG-EV slightly reduced the early apoptosis of MNCs but did not affect late apoptosis, suggesting that EV treatment did not induce cytotoxicity (Figure 2G). Next, we tested the immunosuppressive effect of TSG-EV on proliferation of activated PBMCs. CFSE-labeled PBMCs were co-cultured with EVs in the presence of anti-CD3/CD28 beads and IL-2. CFSE dilution analysis indicated that addition of TSG-EV more potently inhibited total T, CD4^+^ T, and CD8^+^ T cell proliferation than did CTL-EV (Figure 3A, B). The percentage of cells in initial division cycle 1 markedly increased, and the percentage of cells in division cycles 4 and 5 was significantly decreased (Figure 3C) in TSG-EV-treated PBMCs. These results suggest that TSG-primed EVs have a significant inhibitory effect on T cell proliferation.

### 3.4. TSG-Primed EVs Enhance Regulatory T Cells and M2-Type Macrophage Polarization

It has been reported that MSC EVs can mediate immunomodulatory effects through induction of regulatory T cells (Tregs) and M2-type macrophage polarization [20,33]. Thus, we investigated the effect of TSG-EV on specific T cell subsets. Helper T cells (Th) were induced by stimulation with lineage-specific cytokines on CD4^+^ cells purified from human PBMCs in the presence of EVs. Co-culture with TSG-EV significantly reduced the induction of IFNγ^+^CD4^+^Th1 and IL-17A^+^CD4^+^Th17 cells and increased Foxp3^+^CD25^+^CD4^+^Treg cells compared to CTL-EV (Figure 3D and Supplementary Figure S2A). To investigate whether TSG-EV can modulate the phenotype of macrophages, GM-CSF (for M1-type)- or M-CSF (for M2-type)-stimulated primary human macrophages were cultured with EVs in the presence of M1-type cytokines or M2-type cytokines. M1-type macrophages had a round appearance, whereas M2-type macrophages displayed spindle-shaped morphology (Supplementary Figure S2B). The M1-type cell surface markers CD80^+^ and CD86^+^ were significantly reduced in M1-type macrophages cultured with TSG-EV compared to those cultured with CTL-EV. However, the expression of M2-type cell surface markers CD206^+^ and CD163^+^ was increased when cultured with EVs, and this effect was greater in the TSG-EV-treated group (Figure 3E and Supplementary Figure S2C). These results suggest that TSG-primed EVs more effectively induced the M2-type macrophage phenotype than did naive EVs. Thus, TSG treatment to MSCs can generate EVs with improved immunomodulatory properties.

### 3.5. TSG-Primed EVs Significantly Alleviate DSS-Induced Colitis

**Figure 4:**
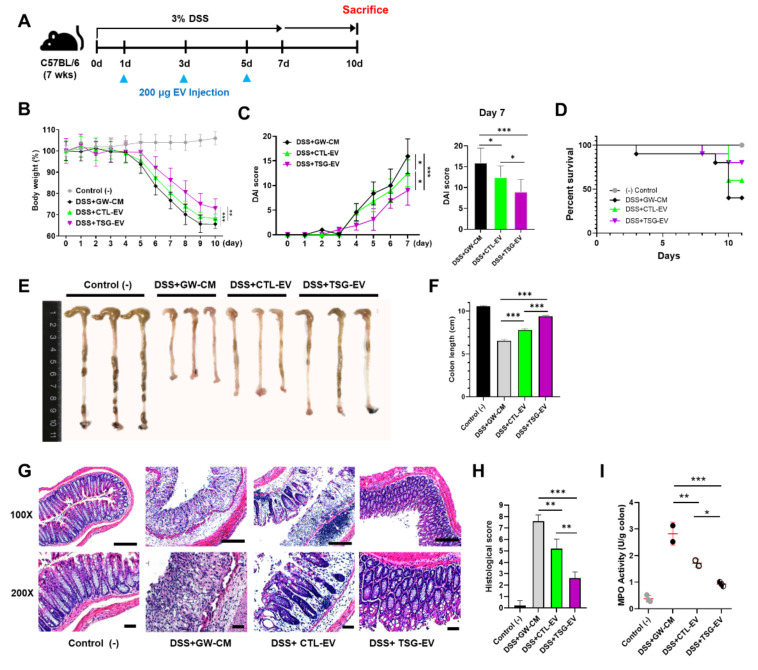
EV from TSG-primed WJ-MSCs ameliorates DSS-induced colitis in mice. CTL-EV and TSG-EV (200 μg in 200 μL PBS) or concentrated-CM derived from GW4869 treated MSCs (vehicle control, 200 μL) were injected intraperitoneally on day 1, 3 and 5 after mice were administered 3% DSS. (**A**) A schematic image of DSS-induced colitis experiment. (**B**). Mice were monitored for changes in (**B**) body weight and (**C**) DAI score (**D**) survival rate. (**E**,**F**) Mice were sacrificed at day 10 and colon lengths were measured. (**G**) H&E staining of colon sections and (**H**) histological scores are shown. Scale bar, 100 μm. (**I**) Neutrophil infiltration was determined by measuring colonic MPO activity on day 10. *n* = 3 mice per group. Results were shown as mean ± S.D. (* *p* < 0.05, ** *p* < 0.01, *** *p* < 0.001)

We investigated the potential protective effect of TSG-EV in a 3% DSS-induced colitis murine model (Figure 4A). As a vehicle control, the culture supernatant of MSCs treated with GW4869, an inhibitor of neutral sphingomyelinase supernatant 2 (nSMase2) that regulates EV secretion, was used (GW-CM). Similar to a previous study [34], 10 μM GW4869 successfully inhibited EV release in MSCs (data not shown). GW-CM-treated colitis mice displayed continuous body weight loss and significantly elevated disease activity indexes (DAI) such as stool consistency, bloody diarrhea, and general activity. However, EV treatment ameliorated body weight loss and DAI, as well as increased the survival rate, especially in TSG-EV-treated group (Figure 4B–D). Moreover, shortening of colon length by DSS administration was significantly improved in TSG-EV-treated mice compared to CTL-EV-treated mice (Figure 4E,F).

Histological examination showed that TSG-EV-treated mice maintained colon tissue integrity; significantly reduced structure destruction, crypt loss, and infiltration of inflammatory cells; and had lower histological score compared to CTL-EV-treated mice (Figure 4G,H). Moreover, myeloperoxidase (MPO) activity, indicative of neutrophil infiltration, was significantly decreased in TSG-EV-treated mice compared to CTL-EV-treated mice (Figure 4I). These results indicate that TSG-primed EVs have enhanced protective activity against intestinal inflammation in DSS-induced colitis compared to those in naive EVs.

### 3.6. TSG-Primed EV Increase Anti-Inflammatory Cytokines in the Inflamed Colon

**Figure 5:**
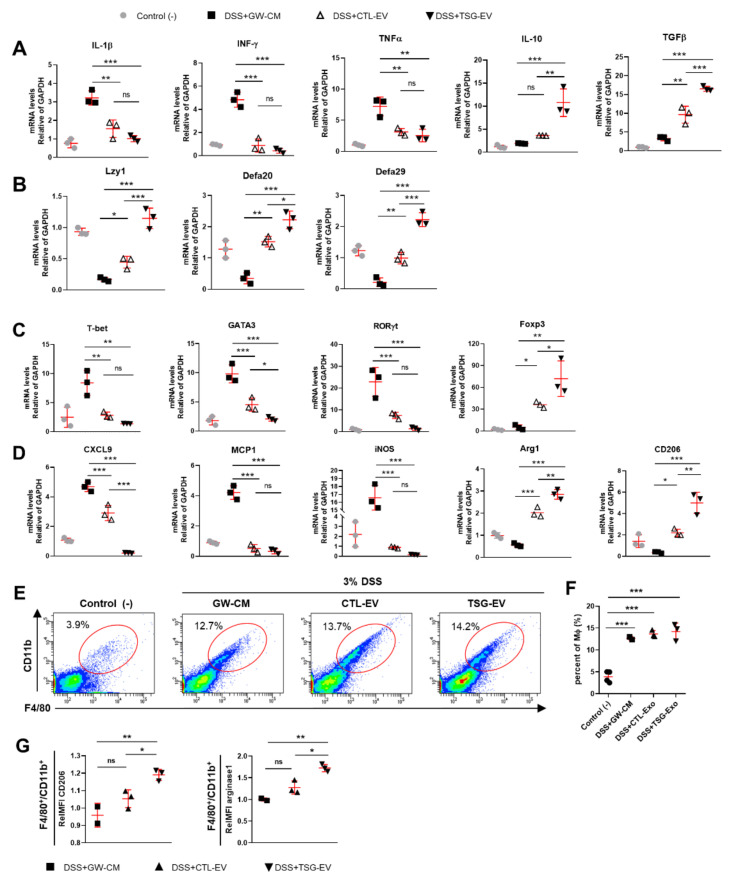
EVs from TSG-primed WJ-MSCs reduce inflammatory response and induce the regulatory T cell and M2 macrophage polarization in vivo. 3% DSS-colitis mice were sacrificed on day 10 and colonic tissue were harvested. mRNA expressions of inflammatory cytokine (**A**) and antimicrobial peptides (**B**) in colonic tissues (*n* = 3 mice per group) were analyzed by qRT-PCR. The expression level of Th1, Th2, Th17 and Treg associated cytokines (**C**) and macrophage phenotype-associated genes (**D**) in colon tissues was evaluated by qRT-PCR. (**E**–**G**) Lamina propria cells were isolated from the colon of 3% DSS-colitis mice (*n* = 2~4 mice per group). The percentage of F4/80^+^CD11b^+^ macrophages in lamina propria cell of colons was analyzed by flow cytometry (**E**) and quantified (**F**). (**G**) Expression of CD206 and arginase1 in F4/80^+^CD11b^+^ macrophages were analyzed by flow cytometry and quantified. The data shows the relative mean fluorescence intensity (RelMFI) normalized to control. Data are shown as mean ± S.D. (* *p* < 0.05, ** *p* < 0.01, *** *p* < 0.001).

Next, we investigated the immune response modulation of EV to attenuate DSS-induced colitis. Expression of pro-inflammatory cytokines, including IL-1β, IFN-γ, and TNFα was decreased in colon tissue of EV treated mice compared to DSS-induced colitis mice, whereas expression of the anti-inflammatory cytokines IL-10 and TGFβ were significantly increased in colon tissue of EV treated mice (Figure 5A). In particular, expression of the anti-inflammatory cytokines IL-10 and TGFβ was potently increased in TSG-primed EV treated mice compared to naive EV treated mice. The abnormal intestinal barrier properties of IBD could affect increased intestinal permeability and inhibit the production of antimicrobial peptides [35]. DSS-induced colitis mice (GW-CM) reduced the expression of antimicrobial peptides in colons tissue, including Lyz1, Defa20 and Defa29, while EV treatment reversed the reduced expression of antimicrobial peptides. Expression of antimicrobial peptides was significantly increased in TSG-primed EV treated mice compared to naive EV treated mice (Figure 5B).

### 3.7. TSG-Primed EVs Enhance Regulatory T Cells and M2 Macrophage Polarization in the Inflamed Colon

Immune cell response plays a crucial role in the pathogenesis of inflammatory bowel disease (IBD). In particular, the balance between Tregs and other T cells in the intestinal microenvironment plays an important role in alleviation of colitis [36]. Therefore, the levels of Th1, Th2, Th17, and Treg cells were assessed in colon tissue of DSS-induced colitis mice. The expression levels of Th1 (T-bet), Th2 (GATA3), and Th17 (RORγt) lineage transcription factors were increased in colon tissue of GW-CM-treated mice but decreased in EV-treated mice. In particular, GATA3 expression was more significantly decreased in TSG-EV-treated mice compared to CTL-EV-treated mice. Foxp3 and Treg transcription factors were significantly increased in TSG-EV-treated mice compared to CTL-EV-treated mice (Figure 5C).

Macrophages play an important role in the pathogenesis of IBD by mediating inflammatory responses through M1/M2 polarization in the colon [37]. Thus, we investigated the in vivo effects of EVs on colonic macrophage polarization. M1-type marker expression of CXCL9, MCP1, and iNOS was decreased in CTL-EV- and TSG-EV-treated mice compared to that in GW-CM-treated mice. CXCL9 level was significantly decreased in TSG-EV-treated mice compared to CTL-EV-treated mice. M2-type marker expressions of Arg1 and CD206 were increased in EV treatment, and this effect was significantly greater in TSG-EV-treated group (Figure 5D). We next investigated the effects of TSG-primed EVs on the immune cell profile of DSS-induced colitis mice. DSS-induced colitis mice showed significantly increased percentage of F4/80^+^CD11b^+^ cells (macrophages) in the *lamina propria* of colons compared to the negative control mice (Figure 5E,F). Treatment of EVs did not affect the percentage of F4/80^+^CD11b^+^ cells (Figure 5E,F), but the expression levels of CD206 and arginase1 in colonic macrophages were significantly elevated in TSG-primed EV-treated mice (Figure 5G). These data suggest that TSG-primed EVs attenuate mucosal inflammation by inducing regulatory T cells and polarizing M2-type macrophages.

## 4. Discussion

During the last decade, our group and others have reported therapeutic properties of MSCs for immune-/inflammation-mediated diseases, including autoimmune uveitis, inflammatory lung diseases, graft-versus-host disease, and liver fibrosis [38,39,40,41]. Although the potential of MSCs as a powerful resource for target diseases is under active exploration in numerous clinical trials, use of MSCs comes with several affiliated concerns, such as difficulty providing a consistent supply with phenotypic/functional stability as well as the high cost of isolation and handling. Furthermore, infusional toxicity, such as acute and transient pulmonary edema, caused by cells lodged in the microvasculature or cellular rejection due to immunological incompatibility has been reported [42]. Immunomodulatory properties of MSCs are mediated by a paracrine effect. EVs play important roles in transporting paracrine factors and in cell-cell micro-communication. Recent studies focused on the immunomodulatory role of MSC-EVs as a therapeutic agent for immune-mediated diseases [43] based on their anti-inflammatory effects by delivering immunomodulatory miRNAs, mRNAs, and proteins to inflammatory immune cells [44]. Lou et al. proposed that MSC-EVs are less immunogenic than parent MSCs because of lower membrane-bound protein content including tetraspanins (CD81, CD63, and CD9), heat-shock proteins (HSP60, HSP70, and HSP90), and TSG 101 [45]. In addition, it has been demonstrated that MSC-EVs do not cause rejection due to activation of allogeneic immune responses in MHC mismatch recipients [15,46] These results suggest that MSC-EVs are a powerful therapeutic tool for future clinical applications. However, regulation of the composition and secretion efficiency of MSC-EVs has not been studied thoroughly. Therefore, appropriate strategies for improving the therapeutic properties of MSC-EVs need to be investigated.

In light of previous studies that demonstrated that ER stress enhances the release of EVs [25,26] and ER stressed-MSCs displayed an effective immunomodulation in RA [27], we tested whether ER stress possibly enhances immunomodulatory properties of MSC-EVs. We evaluated the efficacy and immunomodulatory ability of EVs derived from TSG-primed WJ-MSCs in colitis mice. Although human MSC EVs were administered to a mouse colitis model, no significant immune rejection was observed, suggesting that MSC EVs are immunotolerated by the host even in a xenogeneic system, which is an advantage for future clinical applications. Previous studies showed that several Rab GTPases (Rab7, Rab11, Rab27a, Rab27b, Rab35) and SNARE play a vital role in MVB transport and fusion process, regulating to release of EV [47]. Our results showed that stimulation of WJ-MSCs with TSG significantly increased Rab GTPases (Rab7, Rab11, Rab27a, Rab27b, Rab35) and SNARE expression and EV secretion. In addition, TSG-EV significantly increased the expression level of anti-inflammatory factors such as TGFβ, COX-2 and IDO. Importantly, the expression levels of pro-inflammatory factors (IFNγ, TNFα, and IL-1β) were decreased or remained similar in TSG-EV, suggesting that TSG-induced EV release had a different secretory mechanism from that of inflammaging or senescence-associated secretory phenotype (SASP) of MSCs [48]. Especially, TSG-EV showed ~15 fold higher IDO level compared to CTL-EV, which is a critical regulator of colitis-related inflammation through development of regulatory T cells and M2-type macrophages [6,49]. Furthermore, when TSG-EV were treated to concanavalin A-stimulated MNCs, anti-inflammatory factors from EV were efficiently delivered to MNCs, resulting in similar expression between the EVs and EV-treated MNCs. Because TSG-EV showed superior inhibition of T cell proliferation and better regulation of T cell/macrophage polarization than did CTL-EV, the secretome of EVs might be a reliable indicator for predicting therapeutic functionality of EVs. Although our study suggested enhanced therapeutic effects of TSG-EV with up-regulated anti-inflammatory molecules, further studies need to be performed. First, alteration of microRNAs (miRNAs) expression in EV upon TSG priming needs to be studied as miRNAs are important fraction of EV content and key contributors to the diverse biological function [50]. Therefore, miRNA profiles of TSG-EV and further network analysis may provide an important information to understand the underlying mechanism of TSG-EV therapy. Second, although intraperitoneal injection of TSG-EV showed significant anti-colitic effects, further study needs to be performed to identify the optimal administration route (e.g., intravenous, oral, or subcutaneous) of TSG-EV.

Previous studies have shown that Treg and/or macrophages play an important role in IBD pathogenesis by regulating the function of neutrophils, dendritic cells, and other inflammatory cells [51,52]. In line with previous reports [19,20,53], our data indicated that MSC EVs could modulate macrophage polarization from M1-type to M2-type in the colons of DSS-colitic mice based on significantly enhanced Arg-1 and CD206 expression in colon macrophages. Furthermore, significantly up-regulated Tregs in TSG-EV treated colitic mice possibly played an important role in preventing intestinal inflammation by inhibiting the activity of Th1 and Th17 [36,54,55] or through interaction with macrophages [56,57]. These data suggest that intestinal Tregs and macrophages might be mediators for the anti-inflammatory advantages of MSC EVs. Up-regulation of Tregs and M2-type macrophages maintained intestinal homeostasis as suggested by decreased disease activity score, histological score, and neutrophil activity.

In summary, our findings showed that TSG-mediated ER-stress induction was sufficient to increase the release of WJ-MSC-EVs containing up-regulated anti-inflammatory immunomodulators. Infusion of TSG-EV in experimental colitis significantly enhanced M2-type macrophage polarization and Treg induction in inflamed colons. Our data propose a novel therapeutic strategy for increase the yields of EVs from the MSCs by TSG priming and treatment of colitis based on use of EVs from TSG-primed MSCs.

## Data Availability

The data presented in this study are available on request from the corresponding author.

## Supplementary Materials

The following are available online at https://www.mdpi.com/2227-9059/9/2/209/s1, Figure S1: Characterization of WJ-MSCs and isolation of Exos, Figure S2: Exos from TSG-primed WJ-MSCs regulate T helper (Th) cell differentiation and macrophage polarization, Table S1: Sequences of PCR primers used in this study.
