# Supplementary material for: Extracellular Vesicles from Thapsigargin-Treated Mesenchymal Stem Cells Ameliorated Experimental Colitis via Enhanced Immunomodulatory Properties
Source: Biomedicines. 2021 Feb 18;9(2):209. doi: 10.3390/biomedicines9020209 (PMC7922639; doi:10.3390/biomedicines9020209)

biomedicines

MDPI

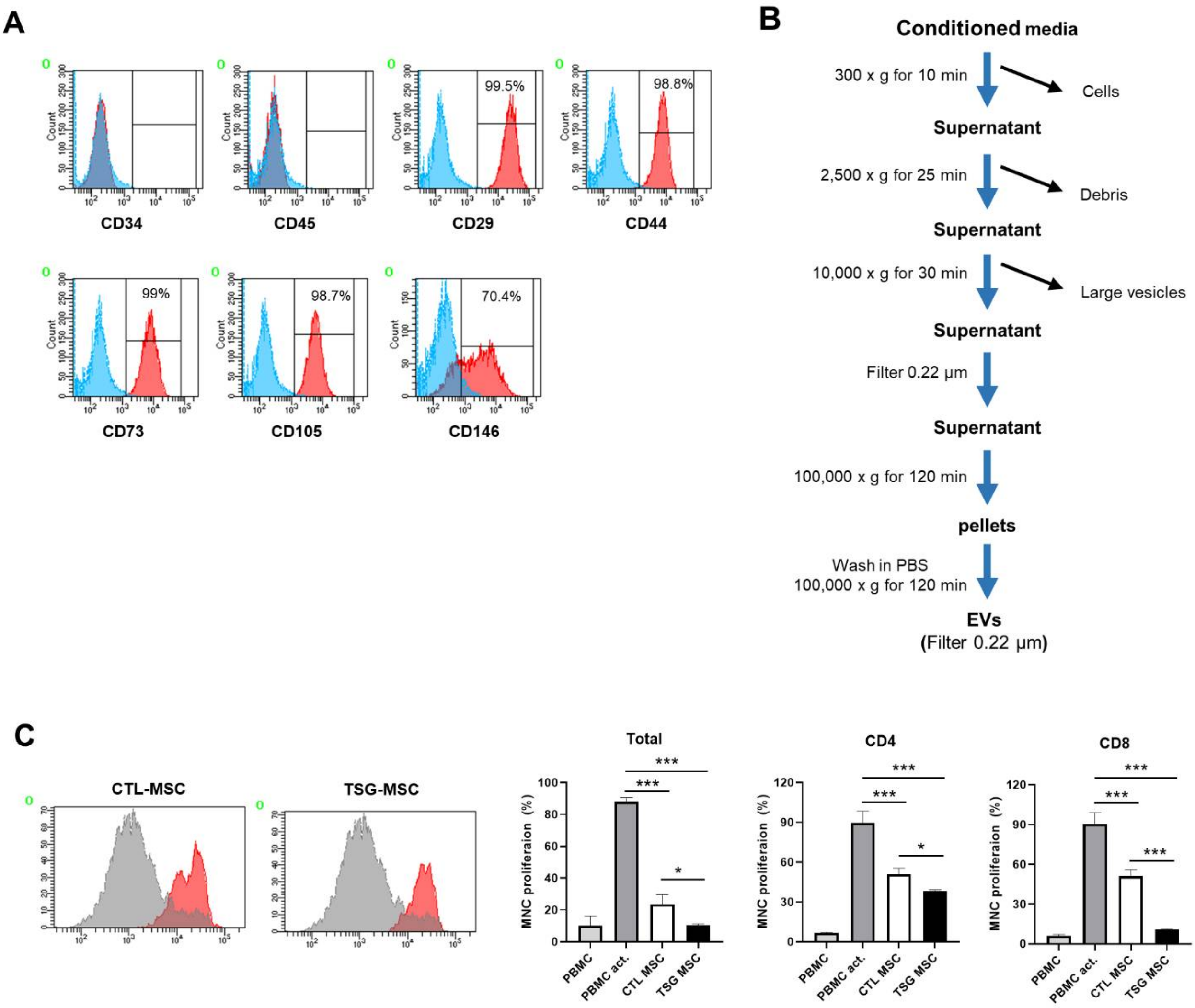

Figure S1. Characterization of WJ-MSCs and isolation of EVs. (**A**) Cell surface markers of WJ-MSCs were measured by flow cytometry for the expression of the MSCs specific antigen CD29, CD44, CD73, CD105, CD146 and CD34, CD45**.** (**B**) Scheme of the ultracentrifugation-based EV isolation. (**C**) CTL-MSCs and TSG-MSCs were co-cultured with CFSE-labeled PBMCs in the presence of anti-CD3/CD28 beads and IL-2. After 6 days, proliferation of T cell was measured by flow cytometry analysis. The data are shown as the mean ± S.D. of three independent experiments (* $p < 0.05$, *** $p < 0.001$).

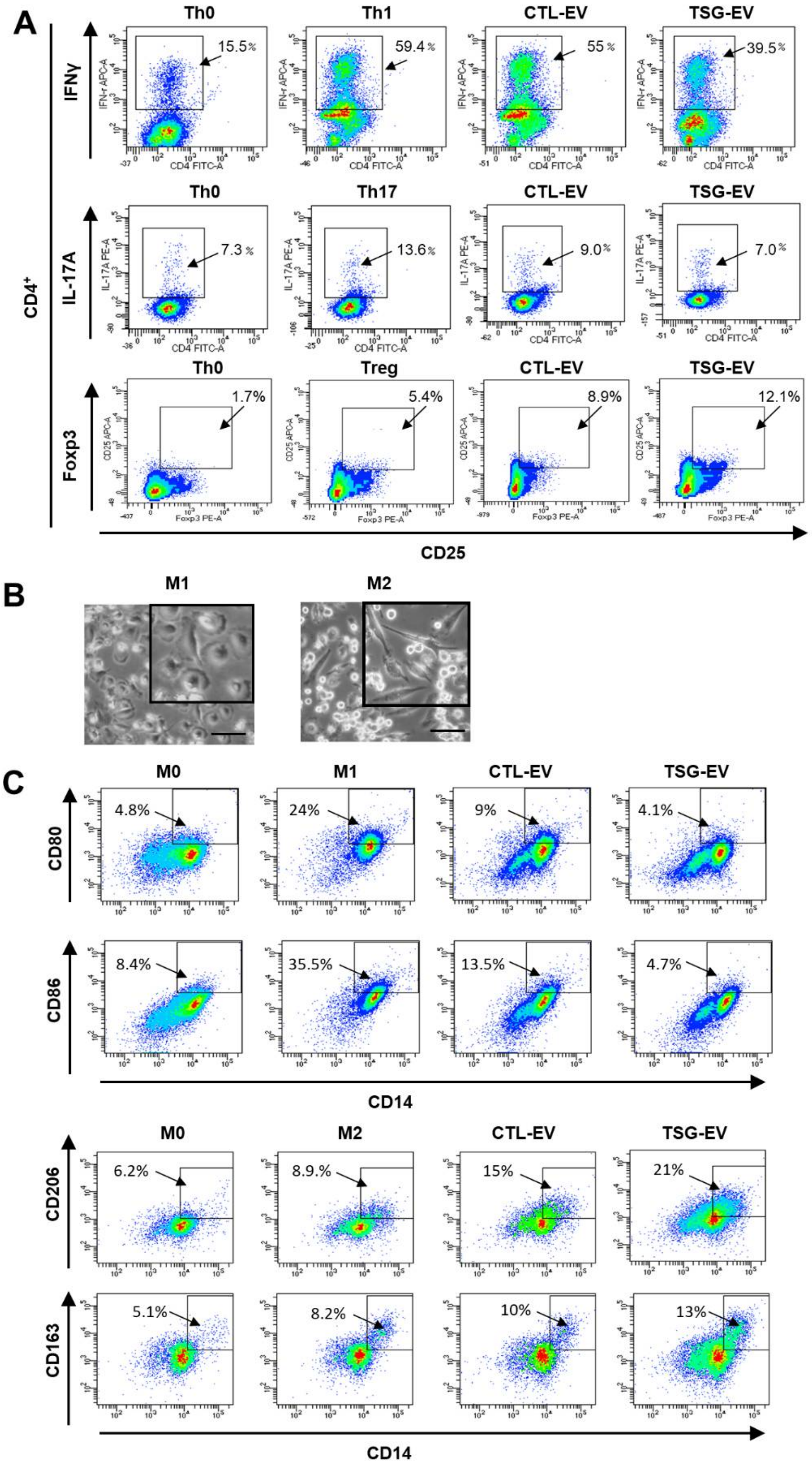

Figure S2. EVs from TSG-primed WJ-MSCs regulate T helper (Th) cell differentiation and macrophage polarization. (**A**) CD4+ T cells were incubated with specific lineage-driving cytokines with or without EVs derived from naive or TSG primed WJ-MSCs in the presence of anti-CD3/CD28 beads and IL-2 for 5 days. The percentage of Th1, Th17 and Treg cells were analyzed by flow cytometry. (**B**,**C**) GM-CSF or M-CSF-induced macrophages were activated with either the M1 cytokines or M2 cytokines for 48 h. (**B**) Representative images of M0, M1, and M2. Scale bar, 100 μm. (**C**) Expression of M1 and M2 macrophage surface markers were analyzed by flow cytometry.

Table S1. Sequences of PCR primers used in this study.

Gene	Forward (5′→3′)	Reverse (5′→3′)
hCOX2	AGACGCCCTCAGACAGCAAA	TCCTGTCCGGGTACAATCGC
hIDO	CCTGAGGAGCTACCATCTGC	TCAGTGCCTCCAGTTCCTTT
hIFNγ	GAGTGTGGAGACCATCAAGGAAG	TGCTTTGCGTTGGACATTCAAGTC
hIL-10	TCTCCGAGATGCCTTCAGCAGA	TCAGACAAGGCTTGGCAACCCA
hIL-1β	CTCTTCGAGGCACAAGGCAC	CAAGTCATCCTCATTGCCACTGT
hNOS2	GCTCTACACCTCCAATGTGACC	CTGCCGAGATTTGAGCCTCATG
hTGFβ	GATGTCACCGGAGTTGTGCG	GCCGGTAGTGAACCCGTTGAT
hTNFα	CTCTTCTGCCTGCTGCACTTTG	ATGGGCTACAGGCTTGTCACTC
hRab27a	GAAGCCATAGCACTCGCAGAGA	CAGGACTTGTCCACACACCGTT
hRab27b	TGGCAACAAGGCAGACCTACCA	CTCCACATTCTGTCCAGTTGCTG
hRab7	GTGATGGTGGATGACAGGCTAG	AGTCTGCACCTCTGTAGAAGGC
hRab11	ACCCCAGCTCTCGATCTCTT	ACATTTCAGTAACGGGCGGG
hRab35	CAGCCCATCTTACTGCAAGCAG	GCTGACAACCTGTCGGAGAGAA
hSNARE	CTGTTAGAGCGAGGTGAGAAGC	GATGGTGCCATTCCAGCATTGG
mArg1	CTGCCAAAGACATCGTGTAC	CTTCCATCACCTTGCCAATC
mCD206	GCAAAGAGAAGGAAACCATG	CCAATAAAATATGGTGACTGCCC
mCXCL9	GTTGTCCACCTCCCTTCGGT	CCAGCTGTCAGATGCAAGGG
mFoxp3	TTGGCCAGCGCCATCTT	TGCCTCCTCCAGAGAGAAGTG
mGATA3	GATCCAGCACAGAAGGCAGG	CGCTTGGGCTTGATAAGGGG
miNOS	GAGACAGGGAAGTCTGAAGCAC	CCAGCAGTAGTTGCTCCTCTTC
mMCP1	CTGGAGCATCCACGTGTTGG	TCCTTCTTGGGGTCAGCACAG
mRORγt	GAAGGCAAATACGGTGGTGTGG	GCTGAGGAAGTGGGAAAAGTC
mT-bet	TTCCCAGCCGTTTCTACCCC	CGTCTTGGCCCCGGTAGTAG

Original image corresponding to Figure 1B.

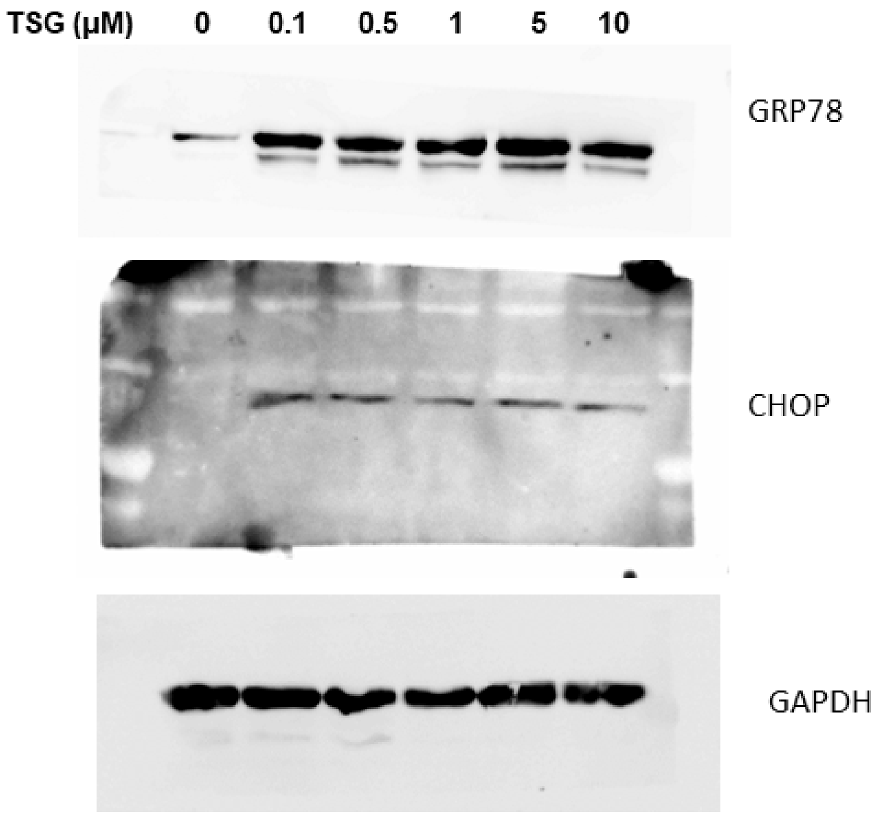

Original image corresponding to Figure 1E.

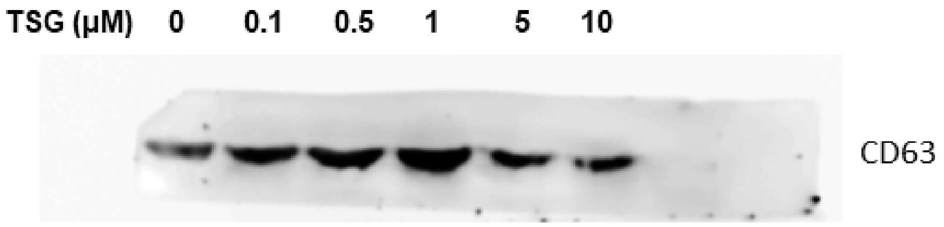

Original image corresponding to Figure 2B.

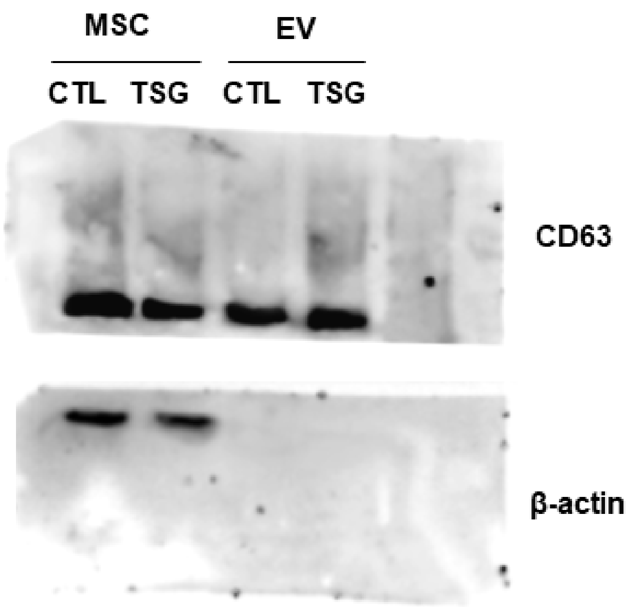

Original image corresponding to Figure 2E.

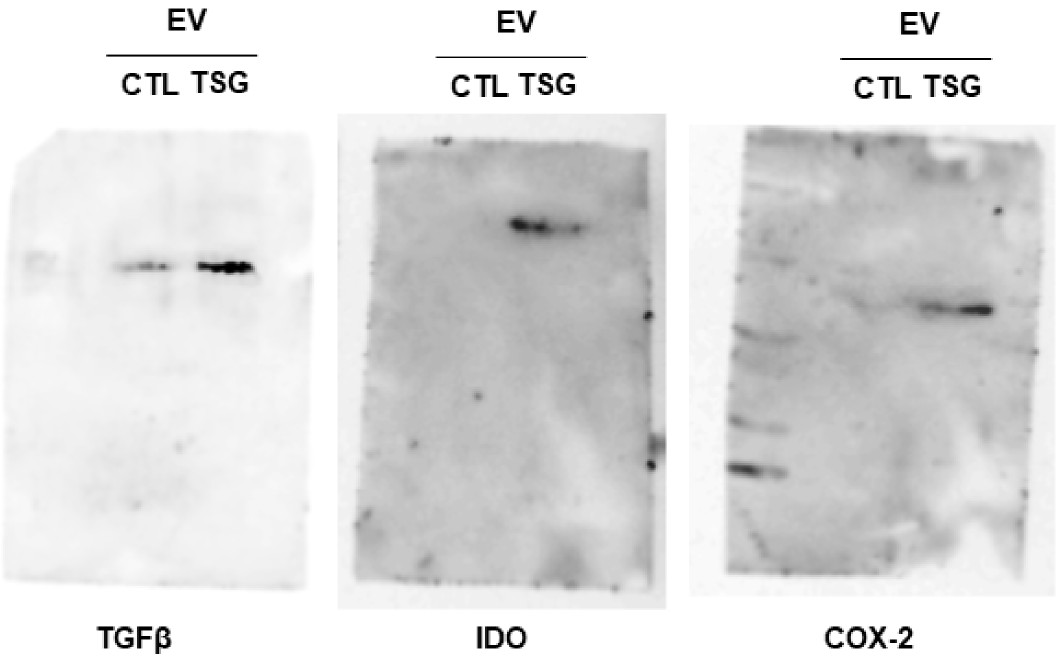

Supplement: Supplementary file 1 [file biomedicines-09-00209-s001.pdf]
